# Factors associated with routine vaccination card retention among children aged 0–59 months in Yaounde-Cameroon: A cross-sectional survey

**DOI:** 10.1371/journal.pone.0273515

**Published:** 2022-08-26

**Authors:** Martin Ndinakie Yakum, Atanga D. Funwie, Atem Bethel Ajong, Marcellin Tsafack, Linda Evans Eba Ze, Ekome Serge Raoul Ekukole, Zahir Shah

**Affiliations:** 1 Department of Epidemiology and Biostatistics, School of Medical and Health Sciences, Kesmonds International University, Cameroon; 2 Department of Biochemistry, Faculty of Science, University of Dschang, Dschang, Cameroon; 3 Medical Department, Doctors Without Borders (MSF-OCG), Yaoundé, Cameroon; 4 Department of Public Health, Faculty of Medicine and Pharmaceutical Sciences, University of Dschang, Dschang, Cameroon; 5 Contract Development and Verification Agency (CDVA)-Performance-based Financing (PBF) for the South West region, Cameroon; Universitas Syiah Kuala, INDONESIA

## Abstract

**Background:**

The routine vaccination card is an important health record for children, but recent findings suggest that vaccination card retention in Cameroon is low, varying from 29%-53%. The aim of this study was to assess factors associated with children’s routine vaccination card retention in Cameroon.

**Methods:**

This cross-sectional survey was conducted in Yaoundé in November 2021, targeting children aged 0–59 months. Participants were selected using a 2-stage systematic cluster sampling in which households were selected by a restricted sampling technique. Data were collected by interviewing the children’s parents/guardians, and a vaccination card was said to be retained if it was presented to the interviewer by the interviewees. Data were analysed using multiple logistics regression with R version 4.1.0 (2021-05-18).

**Findings:**

A total of 529 households were assessed with 361 children aged 0–59 months enrolled: 51% girls and 49% boys. Children aged 0–11 months represented 24.4% of all participants, and children aged 12–59 months were 74.6%. Vaccination card retention was 24% (87), and positive predictors of card retention included: girl child (adjusted Odds Ratio = 1.34, p-value = 0.0269), the respondent being one of the biological parents of the child: mother (adjusted Odds Ratio = 5.97, p-value = 0.0034) or father(adjusted Odds Ratio = 4.69, p-value = 0.0067), and living in a richer household (adjusted Odds Ratio = 1.56, p-value = 0.038). On the other hand, negative predictors of card retention were: child aged 12–23 months (adjusted Odds Ratio = 0.44, p-value = 0.0209) or aged 24-59months (adjusted Odds Ratio = 0.13, p-value = 0.0000), and having an employed mother (adjusted Odds Ratio = 0.34, p-value = 0.0066).

**Conclusion:**

Vaccination card retention in children aged 0–59 months in Yaoundé is low when compared with findings reported by studies from other locations in Cameroon. Besides, the poor and older children have lower odds of keeping routine vaccination cards. There is a need to design interventions to improve vaccination card retention, which considers household wealth and the age of the child.

## 1. Introduction

The importance of immunisation in global health can no longer be overemphasised. The impact of immunisation on morbidity and mortality of specific infectious diseases is enough evidence of immunisation merits. The global eradication of smallpox [1], elimination of poliovirus in all the 6 World Health Organisation (WHO) regions by 2020 [2, 3], elimination of yellow fever disease in some regions [4, 5], and a significant global fall in the incidence of most vaccine-preventable diseases (VPDs), are thanks to immunisation practices [6–8]. According to the WHO vaccine-preventable disease monitoring system summary for 2020, Cameroon recorded a significant reduction in all vaccine-preventable diseases from 1980 to 2019 [9]. Cameroon has not registered any cases of diphtheria, pertussis, and mumps from 2000 to 2021 [9]. Frequently reported barriers to childhood immunisation are grouped under provider/system barriers (cost, access, missed opportunities) and family/social barriers (socioeconomic and vaccine hesitancy) [10]. However, the importance of immunisation data and record keeping is often omitted, despite the crucial role it plays in children’s immunisation schedule follow-up and evidence-based programme decision making. During the ongoing COVID-19 pandemic, childhood vaccination has been significantly disrupted, urging researchers to develop strategies for overcoming the vaccination challenges [11].

In Cameroon, immunisation data are collected at the point of service delivery in vaccination registers and individual vaccination cards [12]. The vaccination register is a paper-based record kept at the level of the health facility and is used to collect immunisation programme data for the estimation of administrative coverage and other indicators [12]. On the other hand, the vaccination card is kept by the parent or guardian of the vaccinated child, for follow-up of the immunisation schedule, and it is used as a reference source at the household level during immunisation coverage surveys [13]. Individual vaccination card is provided for every child receiving their first dose of the routine vaccine (BCG). Henceforth, the parent must bring the card along during each vaccination schedule to be updated by the provider [12]. A child must have a vaccination card before he/she can be administered a vaccine. When the card cannot be found during follow-up visits, the parent, would buy a new card and the health staff will fill it based on health facility records or parent’s recall before figuring out the next vaccine dose that is due to be administered.

Children’s vaccination card is an essential health document for parents, healthcare providers and researchers [14]. Parents use it to track the immunisation status and follow their child’s immunisation schedule. The parents also keep it as documented proof that the child has been vaccinated [15]. Healthcare professionals can quickly consult the individual vaccination card of the child to find missing vaccines, especially when a child is displaced from the original geographic area to another. This helps to ensure the continuity of immunisation services without revaccination or a miss of vaccine doses [16]. Moreover, during household surveys and research, investigators use vaccination cards as proof of vaccination [13].

Based on existing literature, vaccination card retention varies across countries and is particularly low in developing countries [16–19]. Also, vaccination card retention has been reported to be associated with mother’s education and child’s age [16–19]. In Cameroon, vaccination card retention has been reported to be 47% at national level in 2018 [13], 31% in Foumban Health District in 2020 [20], and 52% in Dschang Health District in 2015 [21]. Low vaccination card retention was associated with incomplete immunisation in children in Dschang Health District in 2015 [21]. However, no study has evaluated factors associated with vaccination card retention in Cameroon. The aim of this paper is to assess factors associated with vaccination card retention in children aged 0–59 months in Yaoundé-Cameroon. Yaoundé was selected to have an idea of vaccination card retention in urban area.

## 2. Materials and methods

### 2.1. Ethical approval

The ethical clearance for this study was approved by the Centre Regional Ethics Committee (CE No 01410/CRERSHC/2021). In conformity with the protocol authorised by the ethics committee, verbal consent was obtained from the parents of children before enrolment. The information on the study aims, procedures and the rights of the participants was read out to the parents by the surveyors. The parents were given time to ask questions and allowed to freely decide whether to take part with the child or not. Their consent was simply verbal, and this was preferred to written consent because taking part in the study only exposed them to a minimal risk.

### 2.2. Research design

It was a cross-sectional community-based survey conducted in Yaoundé in November 2021, targeting children aged 0–59 months and their parents/guardians. Participants were selected by a two-stage systematic cluster sampling, with households selected by a restricted sampling technique. Data collection was done by interviewing parents/guardians, and only children whose vaccination cards were presented to the interviewer were considered to have retained their vaccination cards. Data were analysed with R version 4.1.0 (2021-05-18).

### 2.3. Research area

This study was carried out in Yaoundé, the administrative capital of Cameroon. Yaoundé is situated in the Centre region of Cameroon and has an estimated population of more than 2.8 million distributed in six (6) health districts. According to the 2011 update of health facilities in Cameroon, it has 366 health facilities. Yaoundé was selected to have an idea of vaccination card retention in an urban area.

### 2.4. Study population

This study targeted children aged 0–59 months and their parents (or guardians). Though routine immunisation in Cameroon targets children aged 0–11 months, we decided to include all children younger than 5 years old in the study to be able to compare our findings between children still in the immunisation program and those who were supposed to have completed their Expanded Programme on Immunisation (EPI) schedules.

### 2.5. Sample size calculation

A total of 291 participants were needed for this study based on the sample size estimates using the single proportion sample size formula [22]. The parameter used in sample size calculation included: the national level vaccination card retention in Cameroon (47%) [13], the desired precision (d = 7%), the design effect (deff = 1.5), and the 95% confidence level (z = 1.96).

### 2.6. Sampling methods

A total of 30 clusters were selected using systematic sampling with probability proportionate to population size (PPS), using the ENA (Emergency Nutrition Assessment) software version 2021 (https://smartmethodology.org/survey-planning-tools/). Clusters were made up of quarters in the study area, and a list of all quarters(clusters) with their population figures was obtained from the district health services. This list was then introduced in ENA (Emergency Nutrition Assessment) with their population figures to select 30 clusters with PPS. This is a standard method recommended by WHO guidelines to ensure the representativeness of the study population [23]. In each cluster, 24 households were selected using a restricted sampling method. Restricted sampling here refers to a modified systematic sampling in which we randomly select one household within successive sampling intervals. The reason for using this sampling method was to increase the role played by chance in household selection by ensuring that the choice of one household did not condition the selection of the next, as is the case in systematic sampling. All children aged 0–59 months living in each selected household were included in the study.

### 2.7. Data collection

Data were collected using a questionnaire designed by the investigators. The questionnaire had variables on the demographic (gender, age, birth order, place of residence, place of birth) and social characteristics (level of education of parents, employment of parents, marital status of parents) of the child and parents, and selected household characteristics and possessions such as number of people living in one room, type of water source, type of toilet, having a television, having a car, having a motor bike, having a telephone, having a fridge, type of cooking fuel, and type of floor materials, to estimate wealth index (see the study questionnaire (S2. File)). Data collection was done with electronic forms in tablets using KoBo collect (https://www.kobotoolbox.org/). Prior to the start of the survey, interviewers were trained, and tools were pretested and reviewed by the investigators. Parents or guardians of participants were interviewed, and their vaccination cards were requested to confirm retention. In this study, a vaccination card was defined as any document delivered by the healthcare provider, stating the immunisation status of the child in question.

### 2.8. Data management and data analysis

The database was cleaned using MS-Excel 2019 by visually checking for data consistency. Data analysis was done with R version 4.1.0 (2021-05-18).

Households were grouped into 5 different wealth classes using a wealth index, constructed with Principal Components Analysis (PCA) in R. To construct the household wealth index, the first principal component was used to stand for the household’s living standard. The weights for each variable from this first principal component were used to generate a household score [24]. Vaccination card retention was estimated as a proportion with the corresponding 95% confidence interval (CI). Vaccination card was said to be retained if the survey teams saw and consulted any documented proof of the child’s routine immunisation status.

The effect of household wealth level and other socio-demographic factors on vaccination card retention was evaluated using a logistics regression model, with “vaccination card retention” (Yes/No) as the main outcome. Each potential predictor was first assessed with chi-square, and only variables that had a p-value < 0.35 in chi2 analysis were included in the multiple logistic regression model to obtain the Adjusted Odds Ratio (AOR) and adjusted p-value (Ap-value). Without any backward or forward selection, all eligible variables (p-value < 0.35) were included and kept in the model. Our choice of 0.35 as the cut-off was because we believe that demographic and socioeconomic factors relationship with vaccination card retention would be a complex relationship involving several factors. We, therefore, wanted to include many factors in the analysis. The threshold of significance was fixed at p-value < 0.05.

## 3. Results and discussions

### 3.1. Sample description

In total, the data collection teams reached all the 30 clusters planned for the survey, and 529 households were assessed. A total of 361 children aged 0–59 months were enrolled: 51% girls and 49% boys. Children aged 12–59 months made up 74.6% of all participants, and 24.4% were aged 0–11 months.

### 3.2. Vacination card retention

Among the 361 children aged 0–59 months included in the study, 242 (67.04 [61.89–71.82] %) reported to have had a vaccination card, but only 87 (24.10 [19.85–29.81])% could present their vaccination card. In terms of age, 41 (53.95 [42.13–65.45]%) of children aged 0–11 months, 25 (34.25 [23.53–46.28]%) of children aged 12–23 months, and 21 (13.55 [8.59–19.96]) of children aged 24–59 months presented their vaccination cards during the survey. Table 1 shows the potential predictors of vaccination card retention among children aged 0–59 months in Yaounde.

**Table 1:** Assessment of potential factors associated with vaccination card retention in Yaoundé.

Covariate	Bivariate analysis		Multivariate Logistic Regression	
	OR [95% CI]	p-value	AOR [95% CI]	Ap-value
**Gender**				
*Female/Male*	1.34[0.81, 2.20]	0.2537	2.09[1.09, 4.01]	0.0269*
**Age group**				
*12-23months/0-11months*	0.44[0.23, 0.86]	0.0163*	0.38[0.17, 0.86]	0.0209*
*24-59months/0-11months*	0.13[0.07, 0.25]	0.0000*	0.10[0.04, 0.21]	<0.0001*
**Structure of birth**				
*Health facility/ Community*	0.52[0.11, 2.40]	0.4057		
**Birth order**				
*Second or higher/ First order*	0.95[0.55, 1.63]	0.8528		
**Place of residence**				
*Urban area/ Rural area*	0.49[0.13, 1.87]	0.2950	0.99[0.19, 5.24]	0.9909
**Level of education of Father**				
*Primary school/ Never schooled*	0.68[0.20, 2.32]	0.537	1.58[0.33, 7.62]	0.566
*Secondary school/ Never schooled*	1.10[0.39, 3.15]	0.8514	2.14[0.48, 9.60]	0.3192
*High school/ Never schooled*	0.63[0.23, 1.69]	0.3546	1.06[0.24, 4.77]	0.9374
*Higher education/ Never schooled*	0.64[0.25, 1.63]	0.3511	1.24[0.28, 5.41]	0.7761
*Unknown* ^*φ*^ */ Never schooled*	0.55[0.22, 1.36]	0.1973	0.97[0.28, 3.34]	0.9629
**Age of the father**				
*30 years or older/<30 years*	1.87[1.11, 3.15]	0.0190*	0.83[0.38, 1.79]	0.633
**Child living with Father?**				
*Yes/No*	1.40[0.79, 2.50]	0.2527	1.18[0.42, 3.31]	0.7603
Is the father alive?				
*No/Yes*	5.08[0.45, 56.74]	0.1869	11.28[0.56, 228.18]	0.1144
**Employment status of father**				
*Employed/ Unemployed*	1.5578[0.94, 2.58]	0.0845	1.14[0.55, 2.37]	0.7176
**Level of education of Mother**				
*Primary school/Never schooled*	0.98[0.39, 2.45]	0.9637	1.61[0.47, 5.54]	0.4472
*Secondary school/ Never schooled*	0.49[0.21, 1.14]	0.0973	0.47[0.14, 1.59]	0.2257
*High school/ Never schooled*	0.66[0.31, 1.39]	0.271	0.79[0.24, 2.58]	0.6996
*Higher education/ Never schooled*	0.41[0.18, 0.97]	0.0415*	0.47[0.13, 1.75]	0.2587
*Unknown* ^*φ*^ */ Never schooled*	0.43[0.16, 1.12]	0.084	1.23[0.35, 4.77]	0.6929
**Age of the mother**				
*30 years or older/<30 years*	1.29[0.77, 2.16]	0.3292	1.94[0.97, 3.90]	0.0615
Mother living with the child?				
*Yes/No*	332194.56[0.00, 1.00 exp (12)]	0.97		
**Employment status of mother**				
*Employed/ Unemployed*	0.34[0.20, 0.58]	0.0001*	0.36[0.17, 0.75]	0.0066*
**Marital status of the mother**				
*Union(marriage)/ Single*	0.81[0.47, 1.40]	0.4495	0.52[0.20, 1.36]	0.1804
**Household wealth index level**				
*3*^*rd*^*-5*^*th*^ *quintiles/1*^*st*^ *and 2*^*nd*^ *quintiles*	1.56[0.89, 2.75]	0.1235	2.11[1.04, 4.28]	0.038*
**Relationship of the child with the respondent**				
*Mother/ Neither father nor mother*	5.97[2.30, 15.52]	0.0002*	6.34[1.84, 21.80]	0.0034*
*Father/ Neither father nor mother*	4.69[1.32, 16.67]	0.017*	9.74[1.88, 50.44]	0.0067*

NB: only covariates with p-value < 0.35 in the bivariate analysis were included in the multivariate analysis for adjustment.

*p-value ≤0.05(below the threshold of significance).

^**φ**^The level for education of the child’s mother or father was said to be **unkwon** if the person responding for the houshold did not know it and could not reach the concerned to verify.

Our findings are similar with earlier studies in Cameroon and elsewhere in the sense that all show that routine vaccination card retention is low [20, 21]. However, in terms of absolute value, the retention rate in our study is slightly lower than the findings from other studies [20, 21]. The previous national representative survey conducted in 2018 in Cameroon reported that 53% of children aged 11–23 months could present their vaccination cards [13]. Another study in Dschang (West Cameroon) reported that 50.2% of children aged 11–23 months could present their vaccination card [21]. In Foumban (West Cameroon), the vaccination card retention in children aged 0-59months was 29.9% [20]. According to a study in Ethiopia, only 29% of children aged 11–23 months could present their vaccination cards [25]. A study in Karachi (Pakistan), reported similar results, with 33% of children aged 12–59 months presenting their vaccination cards [19]. The differences in the absolute value of the vaccination card retention seen in our study compared to other studies could be explained by the fact that our study was conducted in Yaounde (urban setting), contrary to earlier studies that were either nation-wide surveys or conducted in rural settings. This suggests that vaccination card retention varies slightly across contexts and that the retention rate among rural dwellers might be higher than that in urban dwellers. However, this hypothesis requires further investigation, and if valid, it will be interesting to assess possible causes.

Being a girl, age of the child less than 11 months, mother aged above 30 years old, unemployed mother, higher household wealth level and the respondent being the biological parent of the child were significantly associated with increased odds of having and presenting the vaccination card during the survey (see Table 1). These findings partly corroborate the results of a study in Pakistan which reported that vaccination card retention was significantly associated with the child’s age and overcrowding at the household level [19]. The variation of vaccination card retention with age suggests that parents do not know the importance of keeping the card even when the child has completed the immunisation schedule. On the other hand, the reason gender affects vaccination card retention is not known and requires further investigation. Concerning the mother’s employment status, we believe that mothers who are employed have little time to follow their children’s immunisation and might occasionally delegate this to a third party (say a family member). Consequently, vaccination card quickly get missing as it is handled by two people (the mother, and a third party). During our data collection, parents who did not have their children’s immunisation card advanced several reasons. These included card misplacement, and “card taken to school for children admission in some nursery or primary schools.” Notwithstanding, others said they had the vaccination card but were not willing to go and search for it because they may need to scatter a lot of stuff to get it, or it would take them an exceedingly long time that they were not willing to use.

When a parent does not keep the vaccination card of a child, they may be some consequences to the individual, the health system and hence the health of the society. Without a vaccination card for a child still in the program, the immunisation service provider may not know exactly what the child has received and the missed vaccines [15]. This can happen, especially when the provider is changed or the child is displaced to a different town [21]. Consequently, the child is at risk of unnecessarily repeating some vaccines, thereby exposing the child to potential adverse events following immunisation [26, 27]. Moreover, this leads to wastage of vaccines (which can lead to unnecessary vaccine shortage), disproportionate increase in workload on the provider’s part, and duplicates of the national immunisation data. This exposes the whole immunisation program to misleading conclusions following routine data analysis [16, 28–30].

On the other hand, the child without a vaccination card runs the risk of missing a vaccine dose because the provider might mistakenly think that the child had received it already [16]. In this case, the child stays at risk of infection [29]. Also, this situation, together with vaccine hesitancy and other factors, makes it difficult for the immunisation program to achieve its goal of herd immunity for the population [31–34]. Therefore, it exposes the child in question and the whole society to the risk of outbreaks of vaccine-preventable disease.

The findings in this manuscript are based on a cross-sectional study and hence cannot confirm causality between factors and card retention. Besides, this study registered a high non-response rate compared to similar studies. High non-response can be a potential source of bias. However, the study design was the most appropriate to study the research question, and the findings of this study can be used to guide health interventions and generate hypotheses for further investigation.

Vaccination card retention is, therefore, a major issue in Cameroon’s immunisation program and public health with consequences at the individual, health system and population levels. The retention is affected by the household wealth index, age of the child, age of the mother, and the respondent’s relationship with the child. If mothers/carers are educated on the importance of vaccination card retention during vaccination sessions and the consequences of no retention, it might contribute to increased retention proportion. On the other hand, further research needs to explore why wealthy households turn to keep their children’s vaccination cards more often than the less wealthy group and the feasibility of implementing digital vaccination cards. This could help to design interventions targeting specific groups.

## Conclusions and recommendations

The vaccination card retention proportion in children aged 0–59 months in Yaoundé is 24%. Being a girl, aged 0–11 months, with a mother aged above 30 years of age, living in a household between 3^rd^ -5^th^ quintiles of wealth index, mother unemployed, and the respondent being the biological parents of the child are significantly associated with higher odds of vaccination card retention. Though a few mothers complained that their children’s vaccination card was in school, and others said they had their vaccination cards, but could not present it, vaccination card retention in Yaoundé is extremely low compared to several other places documented in the literature and elsewhere in Cameroon. Based on these findings, we recommend to the Ministry of Public Health that interventions be designed to improve immunisation card retention. For such interventions to be successful, they must be inclusive of the poor and the rich. This has the potential to improve immunisation data quality collected from survey, and ensures better follow-up of children’s immunisation. On the other hand, our recommendation to researchers is to exploit the causes of vaccination card retention disparity between the poor and the rich and to assess the feasibility of setting up a digital vaccination card system in Cameroon. Research can also be conducted to evaluate effective interventions such as incentives on card retention.

## Supporting information

S1 Data Database of manuscript: Factors associated to vaccination card retention. (CSV)

S1 Questionnaire Data collection tool of manuscript: Factors associated to vaccination card retention. (DOC)

## References

[1] Henderson DA. Smallpox eradication. Public Health Rep [Internet]. 1980 [cited 2021 Jun 27];95(5):422–6. Available from: https://www.ncbi.nlm.nih.gov/pmc/articles/PMC1422744/ 7422808 PMC1422744

[2] Adebisi YA, Prisno DEL III, Nuga BB. Last fight of wild polio in Africa: Nigeria’s battle. Public Health in Practice. 2020;1:100043. doi: 10.1016/j.puhip.2020.100043 34173577 PMC7528738

[3] Nasir UN, Bandyopadhyay AS, Montagnani F, Akite JE, Mungu EB, Uche IV, et al. Polio elimination in Nigeria: A review. Human vaccines & immunotherapeutics. 2016;12(3):658–63. doi: 10.1080/21645515.2015.1088617 26383769 PMC4964709

[4] Soper FL. The elimination of urban yellow fever in the Americas through the eradication of Aedes aegypti. American Journal of Public Health and the Nations Health. 1963;53(1):7–16. doi: 10.2105/ajph.53.1.7 13978257 PMC1253857

[5] White JH. The permanent elimination of yellow fever. Journal of the American Medical Association. 1910;55(8):661–2.

[6] Brenzel L, Wolfson LJ, Fox-Rushby J, Miller M, Halsey NA. Vaccine-preventable diseases. Disease control priorities in developing countries. 2006;2:389–412.

[7] Chalmers JD, Campling J, Dicker A, Woodhead M, Madhava H. A systematic review of the burden of vaccine preventable pneumococcal disease in UK adults. BMC pulmonary medicine. 2016;16(1):1–11. doi: 10.1186/s12890-016-0242-0 27169895 PMC4864929

[8] Menzies R, Turnour C, Chiu C, McIntyre P. Vaccine preventable diseases and vaccination coverage in Aboriginal and Torres Strait Islander people, Australia 2003 to 2006. Communicable diseases intelligence quarterly report. 2008;32:S2–67. 18711998 10.33321/cdi.2008.32.30

[9] WHO W| WH. WHO | Immunization Country Profile [Internet]. According to WHO vaccine-preventable diseases: monitoring system summary for 2020. World Health Organization; 2020 [cited 2021 Jun 27]. Available from: https://apps.who.int/immunization_monitoring/globalsummary/countries?countrycriteria%5Bcountry%5D%5B%5D=CMR&commit=OK

[10] Sabnis SS, Conway JH. Overcoming Challenges to Childhood Immunizations Status. Pediatric Clinics of North America [Internet]. 2015 Oct [cited 2022 Jun 13];62(5):1093–109. Available from: https://linkinghub.elsevier.com/retrieve/pii/S0031395515000760 doi: 10.1016/j.pcl.2015.05.004 26318942

[11] Thanapluetiwong S, Chansirikarnjana S, Sriwannopas O, Assavapokee T, Ittasakul P. Factors associated with COVID-19 Vaccine Hesitancy in Thai Seniors. Patient Prefer Adherence [Internet]. 2021 Oct 31 [cited 2022 Jun 13];15:2389–403. Available from: https://www.ncbi.nlm.nih.gov/pmc/articles/PMC8568699/ doi: 10.2147/PPA.S334757 34754180 PMC8568699

[12] Ministry Of Public Health. NORMES ET STANDARDS DU PROGRAMME ELARGI DE VACCINATION DU CAMEROUN [Internet]. 2009 [cited 2021 Jun 27]. Available from: https://docplayer.fr/7296942-Normes-et-standards-du-programme-elargi-de-vaccination-du-cameroun.html

[13] Statistique/INS IN de la, ICF. République du Cameroun Enquête Démographique et de Santé 2018. 2020 Feb 1 [cited 2021 Jun 27]; Available from: https://dhsprogram.com/publications/publication-fr360-dhs-final-reports.cfm

[14] Datta A, Das S, Mog C, Datta S. A Study to Assess the Prevalence of Vaccination Card Retention of Children between 12 to 23 months age by their Parents in a Rural Area of Tripura. IOSR [Internet]. 2016 Sep [cited 2021 Jun 13];15(09):22–5. Available from: http://iosrjournals.org/iosr-jdms/papers/Vol15-Issue%209/Version-1/E1509012225.pdf

[15] Wagner AL. The use and significance of vaccination cards. Hum Vaccin Immunother [Internet]. 2019 Jun 20 [cited 2021 Jun 13];15(12):2844–6. Available from: https://www.ncbi.nlm.nih.gov/pmc/articles/PMC6930106/ doi: 10.1080/21645515.2019.1625647 31157591 PMC6930106

[16] Valadez JJ, Weld LH. Maternal recall error of child vaccination status in a developing nation. Am J Public Health [Internet]. 1992 Jan 1 [cited 2022 Jan 9];82(1):120–2. Available from: https://ajph.aphapublications.org/doi/abs/10.2105/AJPH.82.1.120 1536315 10.2105/ajph.82.1.120 PMC1694427

[17] Miles M, Ryman TK, Dietz V, Zell E, Luman ET. Validity of vaccination cards and parental recall to estimate vaccination coverage: a systematic review of the literature. Vaccine. 2013;31(12):1560–8. doi: 10.1016/j.vaccine.2012.10.089 23196207

[18] Luman ET, Ryman TK, Sablan M. Estimating vaccination coverage: validity of household-retained vaccination cards and parental recall. Vaccine. 2009;27(19):2534–9. doi: 10.1016/j.vaccine.2008.10.002 18948158

[19] Sheikh SS, Ali SA. Predictors of vaccination card retention in children 12–59 months old in Karachi, Pakistan. Oman medical journal. 2014;29(3):190. doi: 10.5001/omj.2014.47 24936268 PMC4052393

[20] Ateudjieu J, Yakum MN, Goura AP, Tembei AM, Ingrid DK, Landry BB, et al. EPI immunization coverage, timeliness and dropout rate among children in a West Cameroon health district: a cross sectional study. BMC public health. 2020;20(1):1–11. 32054484 10.1186/s12889-020-8340-6 PMC7020570

[21] Russo G, Miglietta A, Pezzotti P, Biguioh RM, Mayaka GB, Sobze MS, et al. Vaccine coverage and determinants of incomplete vaccination in children aged 12–23 months in Dschang, West Region, Cameroon: a cross-sectional survey during a polio outbreak. BMC public health. 2015;15(1):1–11. doi: 10.1186/s12889-015-2000-2 26156158 PMC4496879

[22] Suresh K, Chandrashekara S. Sample size estimation and power analysis for clinical research studies. J Hum Reprod Sci [Internet]. 2012 [cited 2022 Jun 13];5(1):7–13. Available from: https://www.ncbi.nlm.nih.gov/pmc/articles/PMC3409926/ doi: 10.4103/0974-1208.97779 22870008 PMC3409926

[23] World Health Organization. World Health Organization vaccination coverage cluster surveys: reference manual. [Internet]. World Health Organization; [cited 2022 Jun 13]. Available from: https://apps.who.int/iris/handle/10665/272820. License: CC BY-NC-SA 3.0 IGO

[24] Tareq M, Abdel-Razzaq AI, Rahman MA, Choudhury T. Comparison of weighted and unweighted methods of wealth indices for assessing SOCIO-ECONOMIC status. Heliyon [Internet]. 2021 Feb 26 [cited 2022 Jul 22];7(2):e06163. Available from: https://www.ncbi.nlm.nih.gov/pmc/articles/PMC7921812/ doi: 10.1016/j.heliyon.2021.e06163 33718635 PMC7921812

[25] Lakew Y, Bekele A, Biadgilign S. Factors influencing full immunization coverage among 12–23 months of age children in Ethiopia: evidence from the national demographic and health survey in 2011. BMC Public Health [Internet]. 2015 Jul 30 [cited 2022 Jan 17];15(1):728. Available from: doi: 10.1186/s12889-015-2078-6 26224089 PMC4520202

[26] Ateudjieu J, Stoll B, Nguefack-Tsague G, Yakum MN, Mengouo MN, Genton B. Incidence and types of adverse events during mass vaccination campaign with the meningococcal a conjugate vaccine (MENAFRIVAC^TM^) in Cameroon. Pharmacoepidemiol Drug Saf. 2016 Oct;25(10):1170–8. 27174237 10.1002/pds.4027

[27] Tsafack M, Ateudjieu J. Improving community based AEFI (Adverse Events Following Immunization) reporting rate through telephone. The Pan African Medical Journal [Internet]. 2015 Nov 12 [cited 2022 Jan 18];22(351). Available from: https://www.panafrican-med-journal.com/content/article/22/351/full 10.11604/pamj.2015.22.351.8368 PMC4779623 26985269

[28] Akhlaq A, McKinstry B, Muhammad KB, Sheikh A. Barriers and facilitators to health information exchange in low- and middle-income country settings: a systematic review. Health Policy and Planning [Internet]. 2016 Nov 1 [cited 2022 Jan 18];31(9):1310–25. Available from: doi: 10.1093/heapol/czw056 27185528

[29] Bloom DE, Canning D, Weston M. The value of vaccination. In: Fighting the Diseases of Poverty. Routledge; 2008.

[30] Mavimbe JC, Braa J, Bjune G. Assessing immunization data quality from routine reports in Mozambique. BMC public health. 2005;5(1):1–8. doi: 10.1186/1471-2458-5-108 16219104 PMC1266378

[31] Paterson P, Meurice F, Stanberry LR, Glismann S, Rosenthal SL, Larson HJ. Vaccine hesitancy and healthcare providers. Vaccine. 2016;34(52):6700–6. doi: 10.1016/j.vaccine.2016.10.042 27810314

[32] Sallam M, Al-Sanafi M, Sallam M. A Global Map of COVID-19 Vaccine Acceptance Rates per Country: An Updated Concise Narrative Review. JMDH [Internet]. 2022 Jan 11 [cited 2022 Jun 13];15:21–45. Available from: https://www.dovepress.com/a-global-map-of-covid-19-vaccine-acceptance-rates-per-country-an-updat-peer-reviewed-fulltext-article-JMDH doi: 10.2147/JMDH.S347669 35046661 PMC8760993

[33] Mardaleta M, Lubis AR, Diantimala Y, Fahlevi H. Determinants of patient behavioural loyalty on primary health centres: Evidence from a cross-sectional study in Indonesia. F1000Res [Internet]. 2022 May 6 [cited 2022 Jun 13];11:440. Available from: https://www.ncbi.nlm.nih.gov/pmc/articles/PMC9111361/ doi: 10.12688/f1000research.110684.2 35615494 PMC9111361

[34] Rosiello DF, Anwar S, Yufika A, Adam RY, Ismaeil MI, Ismail AY, et al. Acceptance of COVID-19 vaccination at different hypothetical efficacy and safety levels in ten countries in Asia, Africa, and South America. Narra J [Internet]. 2021 [cited 2022 Jul 4];1(3). Available from: https://narraj.org/main/article/view/55 10.52225/narra.v1i3.55 PMC10914086 38450212

