# Supplementary material for: Factors associated with routine vaccination card retention among children aged 0–59 months in Yaounde-Cameroon: A cross-sectional survey
Source: PLoS One. 2022 Aug 26;17(8):e0273515. doi: 10.1371/journal.pone.0273515 (PMC9416987; doi:10.1371/journal.pone.0273515)
Supplement: S1 Questionnaire — (DOC) [file pone.0273515.s002.doc]

*Immunization coverage questionnaire*

Date ¦__¦__¦ / ¦__¦__¦ / 2021 Name of district _____________ Name of health area_________________ Name of Village _____________

Team number ¦__¦__¦ cluster number ¦__¦__¦__¦ Household number ¦__¦__¦

| **No** | **Question** | **Options** |
| --- | --- | --- |
|  | **SECTION A: HOUSEHOLDS POSSESSIONS AND FACILITIES** |  |
| A1 | Number of persons sleeping in one room? Report for the room that takes the highest number of individuals | *0= ≥ 5 persons*  *1=< 5 persons* |
| A2 | Source of drinking water for the household | *0=unimproved source*  *1=improved source* |
| A3 | Type of toilet for the household | *0=unimproved toilet*  *1=improved toilet* |
| A4 | Type of floor materials for the household | *0=cement/earth*  *1=tiles* |
| A5 | Type of cooking fuel for the household | *0=wood/kerosene*  *1=gas/electric* |
| A6 | Presence of refrigerator in the household? | *0=N0*  *1=Yes* |
| A7 | Presence of a television in the household? | *0=N0*  *1=Yes* |
| A8 | Do you have a car in the household? | *0=N0*  *1=Yes* |
| A9 | Do you have a motorbike in the household? | *0=N0*  *1=Yes* |
| A10 | Do you have a fixed telephone in the household? | *0=N0*  *1=Yes* |
|  | **SECTION B: CHILD AND PARENT’S RELATED DATA** |  |
| B1 | Sex of the child | *1=Female*  *2=Male* |
| B2 | Age of the child in months |  |
| B3 | Child’s birth order |  |
| B4 | Child’s place of birth | *1=Rural*  *2=Urban/semi-urban* |
| B5 | Child’s place of resident | *1=Rural*  *2=Urban/semi-urban* |
| B6 | Relationship of the respondent with child | *1=father/Mother*  *2=Others* |
| B7 | if not biological parents, how long have you been with the child |  |
| B8 | Level of education of the father | *0=never schooled*  *1=primary school*  *2=secondary school*  *3= high school*  *4= university/ higher education institution*  *5= not known* |
| B9 | Age of the father |  |
| B10 | Father’s resident with the child? | *0=stays in the same household with the child*  *1= stays elsewhere or died already* |
| B11 | Employment status of the father | *0=unemployed*  *1=employed or self-employed* |
| B12 | Level of education of the mother | *0=never schooled*  *1=primary school*  *2=secondary school*  *3= high school*  *4= university/ higher education institution*  *5= not known* |
| B13 | Age of the mother |  |
| B14 | Mother’s resident with the child? | *0=stays in the same household with the child*  *1= stays elsewhere or died already* |
| B15 | Employment status of the mother | *0=unemployed*  *1=employed or self-employed* |
| B16 | Marital status of the mother | *0=single mother(not married or widow)*  *1=union(married or consensual union)* |
| B17 | Does the child have a vaccination card? | *0=No*  *1=Yes*  *9= don’t know* |
